# New Perspective on Dextran Sodium Sulfate Colitis: Antigen-Specific T Cell Development during Intestinal Inflammation

**DOI:** 10.1371/journal.pone.0069936

**Published:** 2013-07-25

**Authors:** Mary E. Morgan, Bin Zheng, Pim J. Koelink, Hendrick J. G. van de Kant, Lizette C. J. M. Haazen, Manon van Roest, Johan Garssen, Gert Folkerts, Aletta D. Kraneveld

**Affiliations:** 1 Division of Pharmacology, Faculty of Science, Utrecht Institute for Pharmaceutical Sciences, Utrecht University, Utrecht, The Netherlands; 2 Centre for Specialised Nutrition, Danone Research, Wageningen, The Netherlands; INSERM, France

## Abstract

CD4+ T cell responses against oral antigens can develop in inflammatory bowel disease (IBD) patients, which may modulate disease. Dextran sodium sulfate (DSS) colitis is commonly used to study IBD, however, it is not considered the best model in which to study T cell involvement in intestinal disease. Our aim was to determine if antigen-specific T cells could be induced during DSS colitis and if they could be detected after disease resolution. To induce antigen-specific T cells, the tracking antigen, ovalbumin (OVA), was administered orally during colitis initiation. Disease severity was monitored, and the antigen-reactivity of CD4+ T cells examined using CD69 expression. While OVA-directed, CD4+ Foxp3+ regulatory T cells could be detected in the spleens of both OVA-treated control and DSS mice, OVA-reactive, CD4+ Foxp3-T cells were only found in the OVA and DSS-treated mice. These results indicate that during DSS colitis T cells develop that are specific against oral antigens, and they are found systemically after colitis resolution. This gives added depth and utility to the DSS model as well as a way to track T cells that are primed against luminal antigens.

## Introduction

Inflammatory bowel disease (IBD) consists of several chronic inflammatory diseases of the gastrointestinal tract of which Crohn’s Disease (CD) and Ulcerative colitis (UC) are the most prevalent. The etiology is largely unknown, but a widely recognized hallmark is abnormal T cell responses towards intestinal bacteria [1]. CD4+ T cells that are responsive to CBir1 (flagellin), oral antigens, enterobacteria and commensal flora [2–6] have been detected. The pathogenicity of these CD4+ T cells has been confirmed in severe combined immunodeficient (SCID) mice after T cell transfer [5,7], and it has been demonstrated that microbiota-specific effector T cells generated during gastrointestinal inflammation are long-lived giving them the potential to lead to chronic inflammation [8]. Furthermore, two of the most widely used drugs for IBD, tumor necrosis factor inhibitors and azathioprine, work, at least in part, via mechanisms that suppress T cell responses [9,10]

A role for T cells in IBD is further supported by genome-wide association studies, which show that T helper type 17 (Th17) cells and regulatory T cells (Tregs) are important for both UC and CD [11]. Th17 cells recruit and stimulate neutrophils via activation of local tissues using interleukin (IL)-17A and IL-17F, and Tregs regulate effector T cells through a variety of mechanisms, both cell-contact dependent and independent, to prevent autoimmunity and maintain peripheral tolerance [1]. The presence of high amounts of Th17 cells and Th17 cell-derived cytokines in the inflamed colon tissue of IBD patients underscores their likely contribution to intestinal inflammation [12].

The possibility of treating IBD by interfering with the development of pathological T cells is enticing. To specifically target T cells, knowledge about their antigen-specificity would be useful as well as information about the triggers that lead to their development. To study adaptive immune responses within colitis, the T cell transfer model of colitis is preferred [13]. In this model, naïve T cells are transferred to an immune compromised host. The caveat of this model is that it relies on a genetically compromised host and an abnormal imbalance of naïve and regulatory T cells that is not found in wild type animals. This model, thus, does not give insight into the immunological processes behind the development of pathological T cells in an, otherwise, healthy animal.

The dextran sodium sulfate (DSS) model of colitis, in contrast to the T cell transfer model, is a robust model of colitis induced by administering dissolved DSS in the drinking water and is inducible in all backgrounds of mice [14]. It also responds to many drugs used to treat IBD, making it highly representative of IBD [15].

DSS is often considered a toxicity model as *in vitro* studies testing the effects of DSS on epithelial cell lines show that direct exposure causes the cell cycle arrest of epithelial cells [16]. However, these *in vitro* studies did not take into account the role of the mucus layer found in *in vivo* conditions. It is now known that DSS causes intestinal mucus to become permeable to bacteria and possibly to other luminal antigens. This would allow bacteria to come into contact with the epithelial layer below [17] and with the transepithelial dendrites of antigen-seeking dendritic cells [18]. This would suggest that the DSS model, instead of being purely a toxicity model, is also modeling mucus loss and the eventual bacterial penetration found during intestinal trauma.

The fact that acute DSS colitis can be induced without the help of T cells, using purely the innate immune system [19], has made it a poor candidate for T cell research. However, it is known that an adaptive immune response does develop, and T cells accumulate at the site of inflammation [20]. Furthermore, certain mouse strains (including C57Bl/6) develop long-term chronic inflammation characterized by substantial neutrophil infiltration that does not subside [21,22]. This suggests a possible role for T cells that do develop during the DSS-induced acute inflammation.

Very little specific research regarding T cell development in the model exists. As it has not been previously published if antigen-specific T cells develop in mice experiencing DSS colitis, our aim was to investigate if CD4+ T cells directed against oral antigens could be found after the resolution of colitis. We found that while healthy mice developed CD4+ T cells that were reactive against the tracking antigen, ovalbumin (OVA), in only the Treg (CD4+ Foxp3+) population, DSS-treated mice developed reactive CD4+ T cells in both the conventional T cell population (CD4+ Foxp3-) and the Treg population. This demonstrates that potentially pro-inflammatory, antigen-specific CD4+ T cells do develop during DSS colitis and that they can be tracked, making the DSS model more useful for T cell research.

## Materials and Methods

### Ethics statement

All experiments were performed in accordance with the guidelines issued by the Dutch ethics committee for animal studies. The protocol was specifically approved by the ethics committee for animal studies of Utrecht University (DEC Number: 2008.II.06.051 and 2010.II.01.013). All efforts were made to minimize suffering.

### Animals

Female C57BL/6 mice and OTII transgenic mice were purchased from Charles River Laboratories (Maastricht, the Netherlands). All mice were used at 8-12 weeks of age and were housed under standard conditions in the animal facilities at Utrecht University.

### Experimental colitis

Experimental colitis was induced in mice by adding 1.5% (w/v) DSS (MP Biomedicals LLC, Illkirch, France) to the drinking water of the mice for 6 days. Mice were sacrificed on either day 7 or 14 after starting DSS, depending on the experiment. Exposure to OVA to develop OVA-directed T cells was accomplished by adding OVA (Sigma-Aldrich, St. Louis, MO USA) to the drinking water (140 µg/ml) during DSS administration.

The Disease Activity Index (DAI) of colitis used in this manuscript is an adaptation of the method introduced by Cooper et al. [22]. It was determined by combining the scores collected from the weight measurement, feces condition and the detectable presence of blood in the feces leading to a score between 0-8 for each mouse. The loss of weight was scored as follows relative to starting weight: 0 = no weight loss, 1 = <5% weight loss, 2 = 5-10% weight loss, 3 = 10-15% weight loss and 4 = >15% weight loss. The feces condition score was scored as follows: 0 = normal, 1 = soft with normal form, 2 = loss of form/diarrhea and 3 = no feces produced. Fecal blood was tested with a Colo-rectal test kit (Axon Lab AG, Stuttgart, Germany). Fecal blood was scored as follows: 0 = no blood, 1 = blood.

### Histological evaluation of colon damage and immunohistochemistry

On day 7 or day 14 (1 day and 8 days after the end of the DSS cycle respectively), colons were excised between the cecum and the rectum and were prepared for histological evaluation. The colon was opened longitudinally, washed in phosphate buffered saline (PBS), placed on a piece of blotting paper, fixed in 10% formalin for 24 hours, routinely embedded in paraffin as swiss-roles and sectioned (5 µm). The damage and infiltration for samples collected on day 14 were blindly assessed after staining sections with H&E. Individual scores were tallied for the proximal colon (characterized by bulges in the colon wall) and the distal colon (the region starting from end of proximal portion stretching to the anus) and combined for a final histological score. Assessments included four pathological criteria: the severity of cellular infiltration (0 = no infiltration, 1 = infiltration between the crypts, 2 = infiltration in the submucosa, 3 = infiltration in the muscularis externa, 4 = infiltration in entire tissue); the extent of cellular infiltration in the region (0 = no infiltration in the region, 1 = < 25%, 2 = 25%-50%, 3 = 50%-75%, 4 = >75%); percent loss of crypts (0 = no damage, 1 = 30% shortening of crypts, 2 = 65% shorting of crypts, 3 = total loss of crypts, 4 = loss of entire epithelial layer) and the extent of crypt loss (0 = no crypt loss, 1 = < 25%, 2 = 25%-50%, 3 = 50%-75%, 4 = > 75%).

For the immunohistochemical T cell staining, colons collected from mice on day 7 were used. Colon sections (5 µm) were deparafinized, dehydrated and treated for endogenous peroxidase activity by incubating with 0.03% H_2_O_2_ in methanol for 30 minutes. Antigen retrieval was performed by boiling the slides for 15 min in a 10mM Tris/1mM EDTA, pH 9.0 buffer. After cooling and being rinsed three times with PBS, the slides were blocked with 5% goat serum (Dakocytomation, Glustrup, Denmark) in 1% bovine serum albumin (BSA) in PBS for 20 minutes at room temperature. Afterwards, the sections were incubated overnight with anti-CD3 antibodies (Dakocytomation) in 1% BSA/PBS for 45 minutes. After thoroughly washing with PBS, the slides were incubated with biotinylated goat-anti-rabbit antibodies (Dakocytomation) and streptavidin-horseradish peroxidase (Vectastain Elite ABC, Vector Laboratories, Burlingame, CA USA) for 45 minutes at room temeperature. Specific binding was detected by incubating the sections with 0.05% diaminobenzidine-tetrahydrochloride (Sigma-Aldrich, St. Louis, MO USA)/0.015% H_2_O_2_/0.01 M Tris-HCL, pH 7.6 for 10 minutes resulting in a brown staining product. Sections were counterstained with Mayers’ haematoxylin (Merck, Darmstadt, Germany), dehydrated and mounted. Slides without primary antibody incubations were included as negative controls.

Photomicrographs were taken with an Olympus BX50 microscope equipped with a Leica DFC 320 digital camera. Contrast of the H&E stained sections was improved and magnification bars were added using Adobe Photoshop CS5.

### Isolation of lymphoid organs and colon cells

Mesenteric lymph nodes (mLNs) and spleens were excised from mice and kept on ice in PBS. To make single cell suspensions, the organs were crushed and the slurry was filtered using 70 µm filters to collect the single cells. The spleens were further purified by destroying the red blood cells by isotonic shock. Cell counts in mLN and spleen were determined using a Coulter Counter (Beckman Coulter, Woerden, the Netherlands). Cells were kept cold until use.

Colon mononuclear cells were isolated from both control and DSS-treated mice on day 7 by incubating the colons with a solution of 0.25 M EDTA for four 15-minute cycles; adding fresh solution after each cycle. The colon tissue was manually disrupted by forcing it through a 70 µm filter. All incubations were performed at 37°C. The mononuclear cells were then isolated by using a 40/90% percoll gradient. Colon mononuclear cells were kept cold until use.

### Detection of memory T cells

Single cell suspensions were first washed with ice cold 1% BSA/PBS. To prevent background staining, cells were first incubated with unlabeled anti-CD16/32 (eBioscience, San Diego, CA USA) for 15 minutes on ice. Cell samples were then stained with antibodies specific for mouse CD3, CD4, CD62L, and CD44 (eBioscience) in the dark, on ice for 30 minutes. After washing with 1% BSA/PBS, the samples were measured with a BD FACSCanto™ II (BD Biosciences, Franklin Lakes, NJ USA). Analysis of the flow cytometry data was performed using BD FACSDiva software (BD Biosciences).

### Intracellular cytokine staining

Single cell suspensions of mesenteric lymph nodes and spleens were cultured in Roswell Park Memorial Institute medium (RPMI, Life Technologies, Paisley, Scotland) supplemented with 1 unit/ml penicillin, 1 µg/ml streptomycin, 50 µM β-mercaptoethanol and 5% FCS in 96 well round-bottom plates at a concentration of 10^5^ cells per well. Intracellular cytokine staining was performed on cells after 24 hours stimulation with purified anti-CD3 (eBioscience) at 2 µg/ml in the medium. Cells were then stained with antibodies for CD4, IFNγ and IL-17A (all antibodies from eBioscience) using the Foxp3 intracellular staining kit (eBioscience). To prevent background staining, cells were first incubated with unlabeled anti-CD16/32 (eBioscience) for 15 minutes on ice as suggested in the manufacturer’s protocol. Fixed samples were kept at 4°C until reading with the flow cytometer. Samples were measured using a BD FACSCanto II flow cytometer. Analysis of the FACS data was performed using BD FACSDiva software (BD Biosciences).

### Serum amyloid A measurement

Serum was collected on day 14 via heart puncture and tested for serum amyloid A (SAA). The SAA ELISA was obtained from Invitrogen (Paisley, UK) and was used according the manufacturer’s directions. The results were read using the Bio-Rad (Hercules, CA USA) iMark™ microplate reader and analyzed using Microplate Manager 6.1 software (Bio-Rad).

### *In vivo* evaluation of antigen presentation

OTII mice were sacrificed and the spleens were removed and kept on ice. Single cell suspensions of the spleens were prepared. The prepared splenocytes were then stained with the vital dye, carboxyfluorescein diacetate succinimidyl ester (CFSE, Molecular Probes, Europe BV, Leiden, The Netherlands). For CFSE staining, cells were suspended in 1% BSA/PBS containing 0.5 µM CFSE and then incubated for 10 minutes at 37°C in the dark. Fetal calf serum (FCS) was then added until an end concentration of 5% was reached. Cells were then washed with 1% BSA/PBS two times before intravenous transfer to mice. 10 million cells were transferred per mouse. After transfer, colitis was induced in the recipient mice by adding DSS (1.5%) to the drinking water for 6 days. On the fifth day of DSS water, mice were administered 400 µg of endotoxin-free OVA (Hyglos GmBH, Bernried, Germany) in saline via oral gavage. Three days later, on day 8, mice were sacrificed and lymphoid organs were removed. *In vivo* proliferation of transferred OTII cells was measured by flow cytometry using a BD FACSCanto II flow cytometer (BD Biosciences). Removed lymphoid organs were prepared as cell suspensions and co-stained with fluorescently labeled anti-CD4 antibodies (eBioscience). Dividing cells were visualized by looking at the dilution of the CFSE intensity of CD4+ lymphocytes. Proliferation of transferred OTII cells was quantified by looking at the percent divided cells within the total CFSE+CD4+ population.

### *Ex vivo* evaluation of T cell antigen-reactivity

Mesenteric LNs and splenocytes were isolated from mice at day 14 of the experiment. Cells were re-stimulated with 25 µg/ml OVA (Sigma-Aldrich) or anti-CD3 (2 µg/ml; eBioscience) and cultured with RPMI medium supplemented with 1 unit/ml penicillin, 1 µg/ml streptomycin, 50 µM β-mercaptoethanol, and 5% FCS in 96 well round-bottom plates at a concentration of 10^5^ cells per well. After 48 hours, cells were harvested and stained with fluorescently labeled antibodies. To prevent background staining, cells were first incubated with unlabeled anti-CD16/32 (eBioscience) for 15 minutes on ice. Cells were first stained extracellularly with anti-CD4 and anti-CD69 and then stained intracellularly for Foxp3. All antibodies and the Foxp3 intracellular staining reagents were obtained from eBioscience. Analysis of the flow cytometry data was performed using BD FACSDiva software (BD Biosciences).

### Statistical analysis

Means with SEM are represented in each graph. Statistical analysis was performed using GraphPad Prism version 5.0 for windows (GraphPad Software, San Diego, CA). Where appropriate, either the unpaired or paired student’s T test or 1-way ANOVA with post-hoc test (Dunnett) were applied. P-values considered as significant are < 0.05.

## Results

### Acute DSS-induced colitis leads to increases in CD4+ central memory T cells

**Figure 1:**
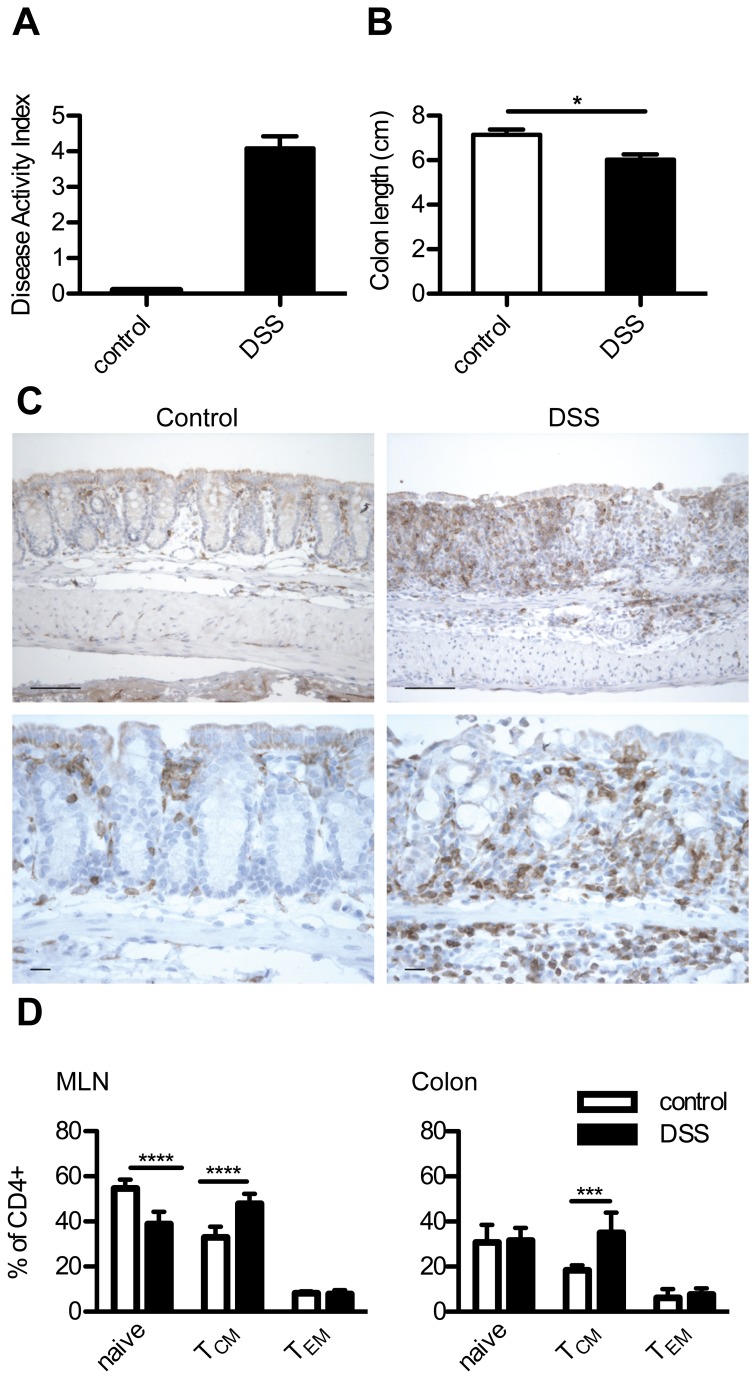
During colitis, T cells accumulate in the inflamed regions of the colon. Mice were treated with DSS for 6 days and sacrificed on day 7. The mice displayed signs of colitis including (A) an increased Disease Activity Index (DAI) and (B) shortened colons. C) Immunohistochemical staining of CD3+ cells in colons obtained from both control (left panes) and DSS-treated (right panes) mice. Top panes are 200x (bar: 5 µm) and bottom panes are 400x magnification (bar: 1 µm). Increased CD3+ cells are observed in inflamed colons. D) Naïve (CD4+ CD62L+ CD44-), central memory (T_CM_, CD4+ CD62L+ CD44+) and effector memory (T_EM_, CD4+ CD62L-CD44+) T cells were measured in the mLNs and colon mononuclear cell suspensions using flow cytometry. Results are expressed as mean + SEM, N = 4-6 mice per group. *** P < 0.001; **** P < 0.0001.

To learn more about the adaptive immune response during colitis, we induced acute DSS colitis in mice. As expected, the colitis symptoms peaked at 7 days after the start of DSS (Figure 1A), and the colons were significantly shortened (Figure 1B). Immunohistochemical staining for CD3 in the colons revealed that T cells collected in the inflamed areas of the colon (Figure 1C). To characterize the activation states of the cells, flow cytometry was used to determine the relative percentages of naïve (CD4+ CD62L+ CD44-), central memory (T_CM_, CD4+ CD62L+ CD44+) and effector memory CD4+ T cells (T_EM_, CD4+ CD62L-CD44-) in colon mononuclear cells and mLN cells collected from healthy and DSS-treated mice. In the colon and the mLN, naïve CD4+ T cells were the most abundant, comprising 30%-60% of the CD4+ T cells. During inflammation, naïve T cells were significantly decreased in the mLN (P < 0.0001, Figure 1D). In healthy colons and mLNs, CD4+ T_CM_ cells comprised 20%-40% of the population. During colitis, CD4+ T_CM_ cells increased significantly in both the colons (P < 0.001) and the mLNs (P < 0.0001). CD4+ T_EM_ cells, the least abundant population, comprised approximately 10% of CD4+ T cells (Figure 1D). Changes in the CD4+ T_EM_ cell population magnitude were not apparent in the colons or mLNs.

### Th17 cells are detected in the spleen after resolution of DSS-induced colitis

**Figure 2:**
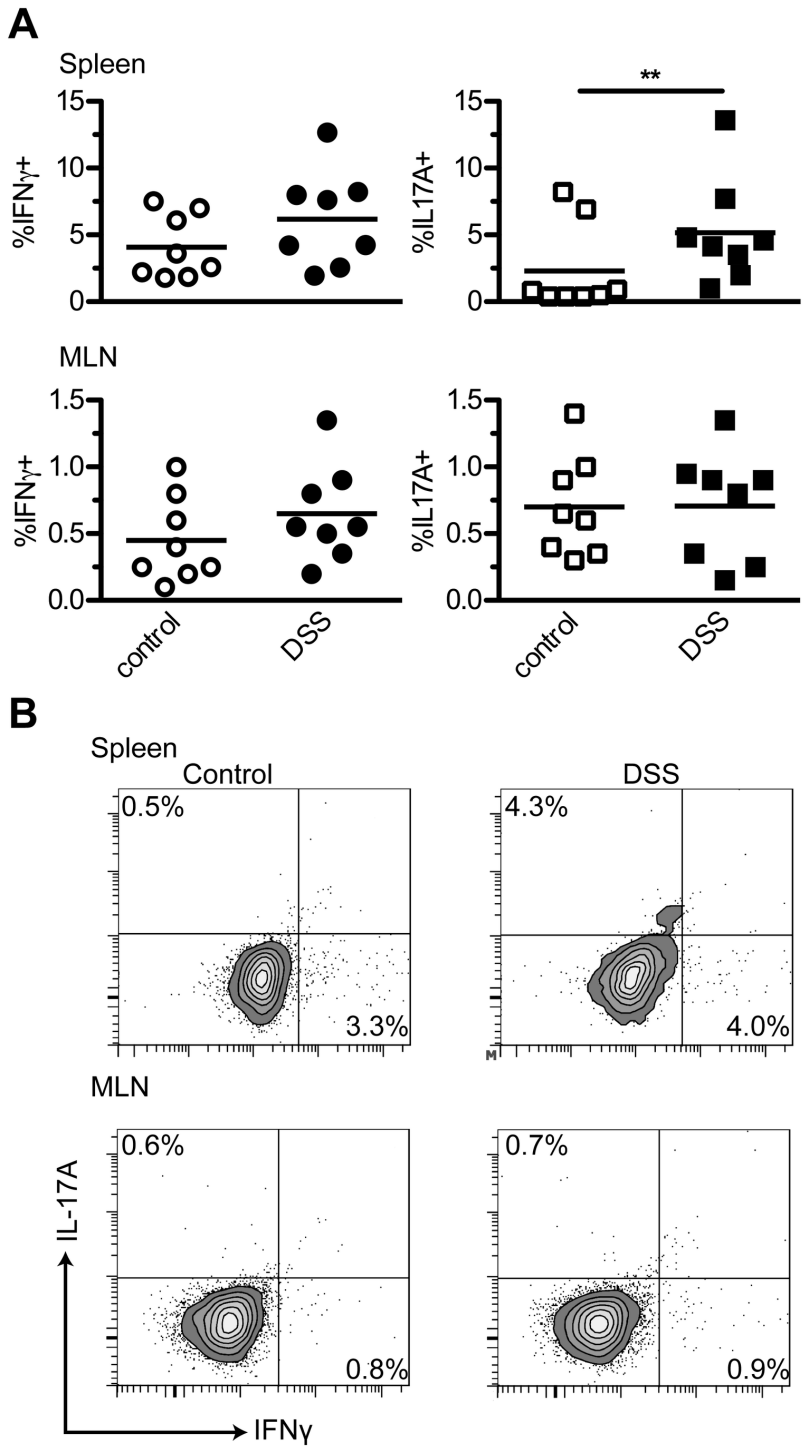
Th17 cells are detected in the spleen after colitis resolution. IFNγ and IL-17A producing CD4+ T cells were detected in the spleens and mLNs, 14 days after the start of DSS using intracellular cytokine staining. A) Percentages depicted are the populations of cytokine expressing CD4+ cells within the total CD4+ population. Bars indicate the mean, N = 8 mice per group. ** P < 0.01. B) Representative FACS contour dot plots for spleen and mLN showing intracellular staining of IL-17A and IFNγ within the gated CD4+ T cell population. Percentages within CD4+ T cell population are shown.

As increased central memory T cells were formed as a result of DSS colitis, the possibility existed that pro-inflammatory T cells could be found in the lymphoid organs of DSS-treated mice after acute disease resolution. Thus, cell suspensions of isolated mLNs and spleens from control and DSS-treated mice were activated with anti-CD3. Intracellular cytokine staining for IFNγ, IL-17A, IL-4 and IL-10 was performed 24 hours later. Increased IL-10 and IL-4 producing CD4+ T cells were not observed (data not shown). However, CD4+ T cells isolated from spleens of DSS-treated mice produced more IL-17A as compared to healthy spleens after anti-CD3 stimulation (P < 0.01; Figure 2A and B). Although suggestively increased in both tissues, IFNγ was not significantly raised. This shows that after the resolution of acute colitis, during the chronic phase of DSS colitis, Th17 cells can be detected in the spleen.

### Oral OVA is taken up from the colons from both healthy and DSS-treated mice

**Figure 3:**
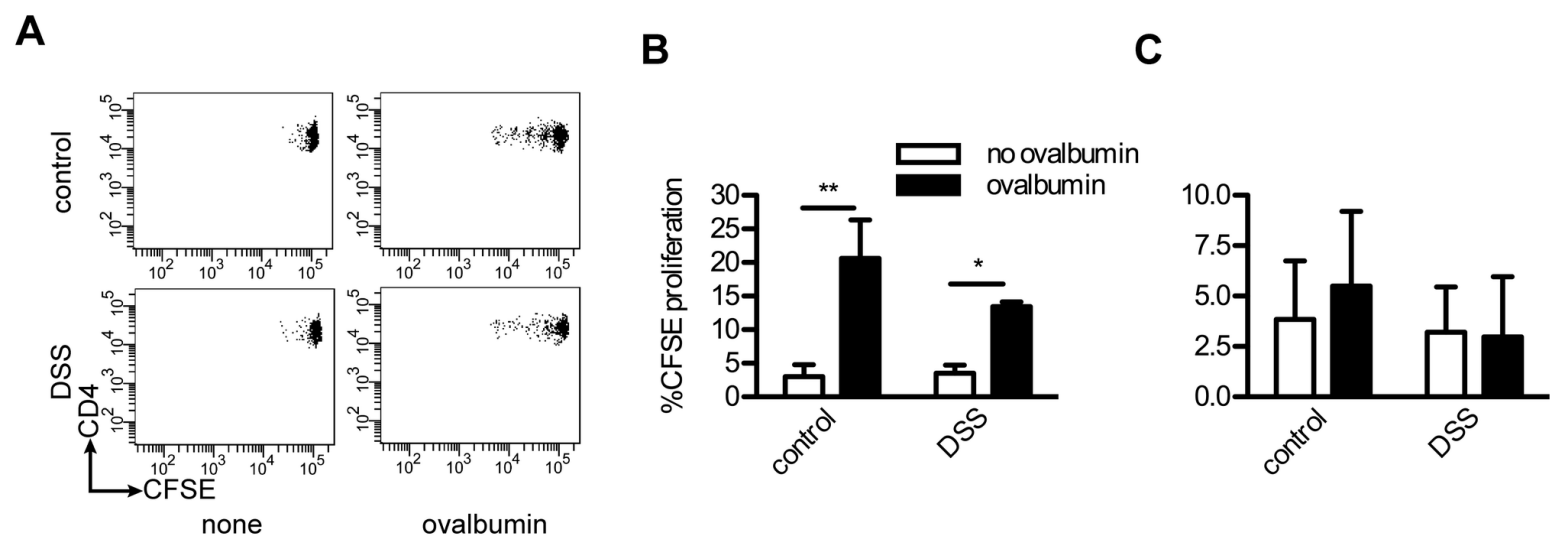
Oral antigens are presented in the draining lymph nodes of both healthy and DSS treated mice. A) OVA presentation in the gastrointestinal tract was visualized by the proliferation of adoptively transferred, CFSE labeled, OTII T cells. Representative FACS dot plots displaying OTII T cell proliferation within the mLNs of both healthy and DSS-treated mice after oral gavage of saline, “no antigen” or oral gavage with OVA, “ovalbumin”. The loss of CFSE intensity is an indication of dividing T cells. B) Percent proliferated OTII cells found within isolated mLNs. C) Percent proliferated OTII cells in the non-local, axillary lymph nodes. Results for (B) and (C) are expressed as mean + SEM, N = 4 mice per group, pooled from two independent experiments. * P < 0.05; ** P < 0.01.

The development of antigen-specific T cells depends on the presence of antigen presenting cells. Oral antigen is taken up predominately by dendritic cells (DCs) in the intestinal tract and presented to T cells in the Peyer’s patches and in the draining lymph nodes, such as the mLNs [23]. The efficiency of oral antigen presentation has not been investigated during DSS-induced colitis. To be certain that ingested OVA would be properly presented within healthy and inflamed intestinal tracts, CFSE-labeled OTII cells were adoptively transferred into mice two days before DSS induction. Transgenic OTII mice have CD4+ T cells with T cell receptors (TCRs) specific for an OVA epitope presented in the context of the murine MHC class II molecule, IA^b^. Three days after oral exposure of OVA, both DSS-treated and healthy mice displayed expanded CD4+ T cells in the mLN (Figure 3A). The percentages of proliferated CFSE-labeled cells were significantly higher in mice given OVA than in the controls for both DSS-treated and healthy mice (P < 0.01 and P < 0.05 respectively, Figure 3B). This indicates that antigen-presenting cells in DSS-treated mice took up oral antigens in the gastrointestinal tract and efficiently presented them during inflammation in a manner similar to healthy mice. To control for spontaneous proliferation of the OTII cells, CFSE positive cells were also examined in non-local lymph nodes (axillary lymph nodes), which would be less likely to come in contract with orally ingested antigen. T cell proliferation was not observed in the axillary lymph nodes (Figure 3C).

### Oral OVA does not change clinical parameters of DSS-induced colitis

In IBD patients, responses to orally administered antigens are measured in the peripheral blood mononuclear cells, 1-2 weeks after the oral feeding of antigens [2]. To provide oral tracking antigens during DSS colitis, we administered OVA via the drinking water along with dissolved DSS for 6 days. The expectation was that oral OVA would not influence the clinical parameters. However, as oral tolerance induction against bystander antigens has been known to result in the amelioration of chronic inflammatory disease models in a phenomenon called bystander suppression [24,25], we monitored the DSS colitis phenotype closely.

**Figure 4:**
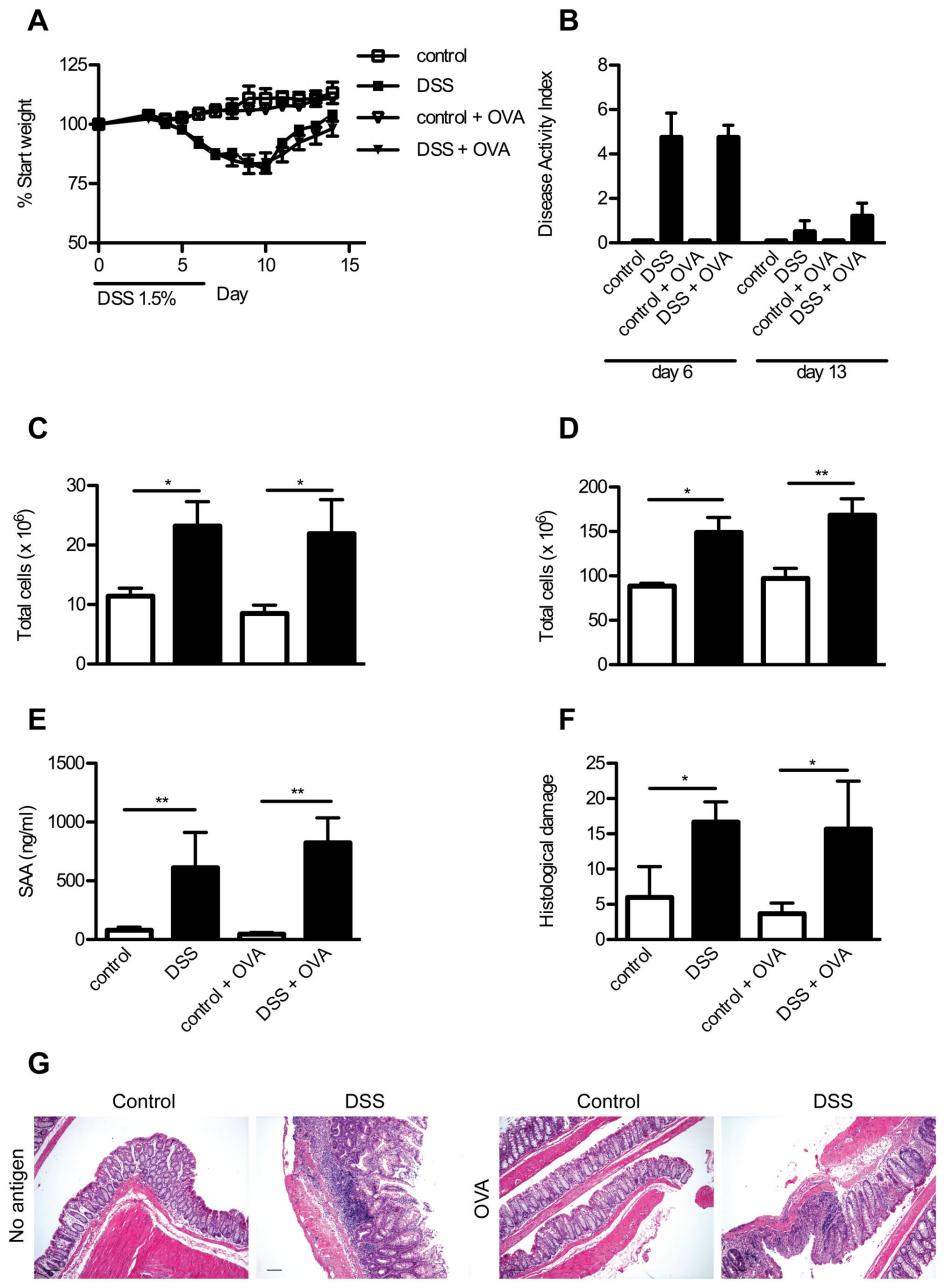
DSS and OVA treated mice have the same clinical phenotype as mice treated with DSS alone. Mice were treated with both DSS and OVA for 6 days, and several clinical parameters were measured during disease progression and after sacrifice. A) Percent weight gain relative to starting weight was measured in each individual mouse for 14 days. B) DAI was calculated on day 6 and day 13 for all groups as described in the materials and methods. N = 10-15 mice before day 7, N = 5-10 after day 7, mice are pooled from two independent experiments. Mesenteric LNs C) and spleens D) were obtained from mice sacrificed on day 14. Cell suspensions were prepared and total cells counted in each organ. Samples (N = 5) were pooled from two independent experiments. E) SAA was measured in the serum from mice sacrificed on day 14. Samples (N = 5) were pooled from two independent experiments. F) Several colons collected from mice on day 14 were examined and scored for histological damage parameters as described in the materials and methods (N = 3, from a single experiment). G) Representative photos are shown for colonic sections from control, DSS-treated, OVA treated controls and OVA and DSS-treated mice. Bar is 5 µm. All graphical results are expressed as mean + SEM in the bar graphs. * P < 0.05; ** P < 0.01.

There was no indication that OVA treatment led to lessened or worsened colitis severity, as judged by the investigated clinical parameters (Figure 4A–G). The weight loss (Figure 4A) and DAI (Figure 4B) measured on day 6 and 13 were normal for the DSS colitis model despite OVA in the drinking water. The total amount of cells in the mLNs (Figure 4C) and spleens (Figure 4D) was similarly increased by DSS treatment in both OVA-treated and untreated animals during sacrifice on day 14. The acute phase protein, SAA, was found to be similarly increased in the day 14 sera from both DSS-treated groups compared to control groups (Figure 4E). Histological analyses of the colons revealed no obvious differences in gross damage or general cellular infiltration between the groups (Figure 4F and G). Both the OVA-treated DSS group and the untreated DSS group had colons with apparent cellular infiltrations, crypt damage and edema (Figure 4G). No differences were detected either clinically or microscopically as a result of adding OVA to the drinking water during DSS colitis.

### Oral OVA does not change the CD4+ T cell subset composition

**Figure 5:**
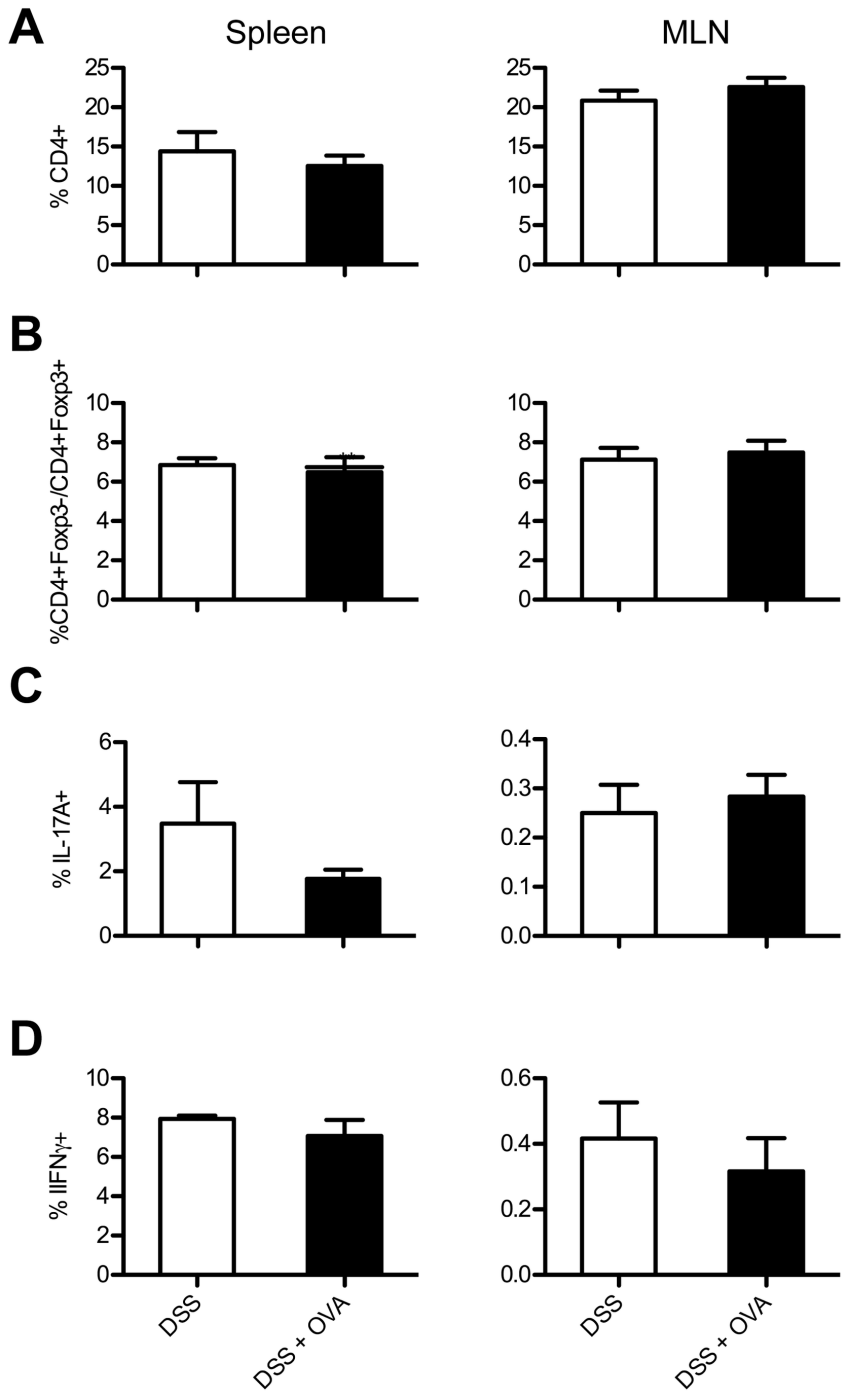
DSS and OVA treated mice have aspecific T cell responses similar to mice treated with DSS alone. Mice were either treated with DSS alone or with DSS and OVA. Spleens and mLNs were removed, stimulated and examined by flow cytometry. A) Percentages of CD4+ T cells were determined within cultures after stimulation with anti-CD3 for 48 hours. N = 4-9 mice per group. B) The ratios of the percentages of Foxp3- and Foxp3+ cells (Foxp3-/Foxp3+) were determined within the CD4+ T cell population. N = 4-9 mice per group. Using intracellular cytokine staining, percentages of IL-17A+ (C) and IFNγ+ cells (D) within the CD4+ cell population were determined after stimulation. N = 3 mice per group. All graphical results are expressed as mean + SEM in the bar graphs.

To determine if the general CD4+ T cell reactivity and subset composition had changed as a result of OVA and DSS in the drinking water at one week after disease resolution, the percentage of CD4+ T cells, the ratios of CD4+ Foxp3-/CD4+Foxp3+ T cells and percentages of IL-17A+ and IFNγ+ T cells were determined in spleen and mLN cells after stimulation with anti-CD3. As shown in Figure 5A, administering OVA together with DSS did not change the percentage of CD4+ found in the samples as compared to DSS alone. Nor did it alter the ratios of CD4+ Foxp3- (conventional T cells)/CD4+ Foxp3+ (Tregs; Figure 5B). Moreover, the percentages of IL-17A+ and IFNγ+ CD4+ T cells in the mLNs and spleens after *ex vivo* stimulation remained unchanged after the addition of OVA to the DSS model.

### Increased percentages of OVA-reactive splenic CD4^+^ Foxp3-T cells are detected in DSS and OVA treated mice after resolution of colitis

**Figure 6:**
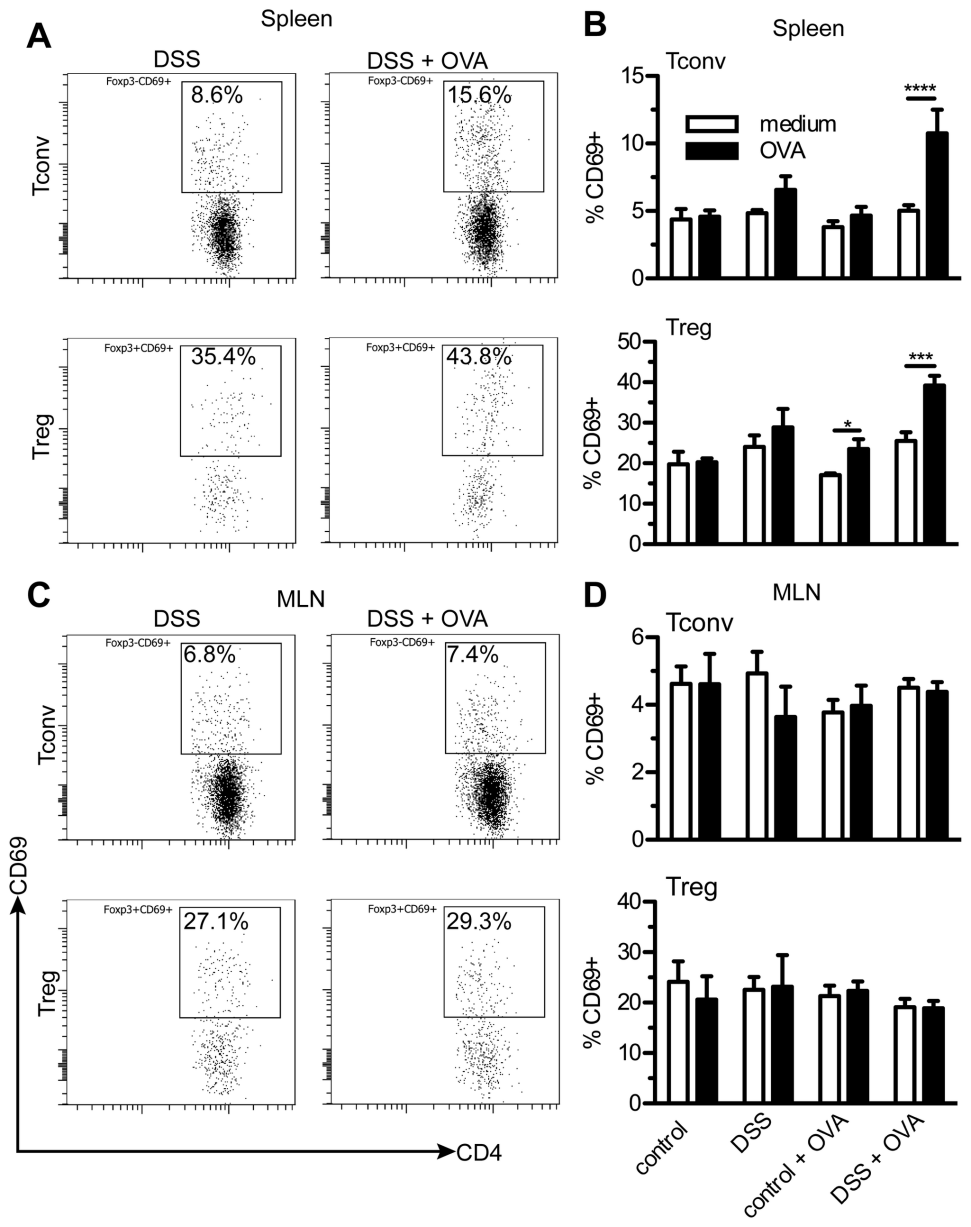
OVA-directed, CD4+ Foxp3-T cells are detected only in mice treated with DSS and OVA. Both mLN and spleen cell suspensions were prepared 14 days after the start of the DSS and OVA treatment for all groups. Cells were stimulated with OVA, and CD69 expression was measured using flow cytometry. A) Representative FACS dot plots of the CD69 expression in CD4+ Foxp3- (Tconv) and CD4+ Foxp3+ (Treg) T cells in the spleen. Percentages shown are the % CD69+ cells in the specific gated Tconv or Treg populations. B) The mean percentage CD69 expression was calculated for Tconv and Treg in the spleen cells of each group. C) Representative FACS plots of the CD69 expression in Tconv and Treg cells in the mLN. D) The mean percentage CD69 expression was calculated for Tconv and Treg cells in the mLN. Results are expressed as mean + SEM, N = 6-9 per group. *** P < 0.001; **** P < 0.0001.

To determine if the splenic CD4^+^ cells were reactive to oral antigens, CD4+ cells from the mLNs and spleens were examined one week after colitis resolution in mice that were administered OVA. Cell suspensions from mLNs and spleens were restimulated with OVA and examined for the expression of the very early activation marker CD69. As shown in Figure 6A and B, OVA-reactive T cells were found in the splenic CD4^+^ Foxp3+ (Treg) populations of both mice treated with DSS and OVA and the OVA-treated healthy controls. However, mice treated with DSS and OVA uniquely had significantly increased percentages of splenic, OVA-reactive CD4+ Foxp3-T cells (conventional T cells). Reactive cells were not observed in the mLNs (Figure 6C and D), and no OVA-directed cells were detected in the control mice not administered OVA in the drinking water (Figure 6A–D). Taken together, these results indicate that antigen-specific CD4+ Foxp3-T cells develop during active colitis, and that they can be found in the spleens of mice after DSS resolution. Moreover, this implies that OVA can be used as a T cell tracking antigen within DSS colitis. 

## Discussion

Research looking at the role of CD4+ T cells in IBD has shown that IBD patients develop T cells that are directed towards luminal antigens like flagellin, intestinal bacteria and oral antigens [2–6]. This information, often the result of examining patient CD4+ T cell clones, does little to explain the actual function of CD4+ T cells in promoting IBD chronicity and how they are generated. *In vivo* models of IBD can be helpful for this research. Lodes et al. justified their finding of anti-CBir1 (flagellin) antibodies and CBir1-specific CD4+ T cells in CD with an *in vivo* experiment. They found that transferring CBir1-specific CD4+ T cells to SCID mice led to the development of colitis, while CD4+ T cells with a normal repertoire of TCRs did not, indicating that antigen specificity is of importance in CD4+ T cell-mediated colitis [5]. Moreover, a study of antigen specificity of CD4+ T cells isolated from SCID mice suffering from colitis after transfer of naïve T cells showed that these cells were highly reactive to fecal extracts and are Th1 skewed [7]. These studies underscore the importance of the T cell transfer model of colitis for intestinal CD4^+^ T cell research. However, because they are induced in SCID mice, which lack functional T and B cells, they are not the best representatives of human IBD and do not allow the study of the immunological processes that lead to the generation of IBD-inducing T cells.

Looking for new alternatives, we chose to use the DSS model of colitis to determine if T cells reactive against luminal antigens could be developed *in vivo* in an experimental colitis model using wild type mice. The DSS-induced colitis model is advocated as a highly relevant model for IBD, being sensitive to common IBD therapeutics [15], sharing a similar gene expression as IBD [26] and displaying T cell accumulation in the inflamed colon [20,27] similar to what is found in IBD patients [28]. Furthermore, many have observed a chronic pathology that develops after the acute inflammation has passed, which includes changes in crypt morphology with lymphocytosis and a Th1/Th2 cytokine profile [14,21,22,29].

This chronic pathology could be caused by memory T cells. Memory T cells are known to function as sentinels of the immune system and often reside in the periphery [30]. During DSS-induced inflammation, tertiary lymphoid structures that are adjacent to the intestinal epithelial layer develop [31], which likely house resident memory T cells. We found increased numbers of T_CM_ cells in our colon mononuclear cell suspensions of our DSS-treated mice. T_CM_ are differentiated mainly on the expression of CD62L, an adhesion molecule that allows them to enter and stay in lymphoid tissues like colon patches. Increased T_CM_ in the colon during DSS colitis could be responsible for the chronic colitis pathology later found in mice [21]. T_CM_ are known to regain effector functions and expand when they re-encounter their cognate antigens [32], which would lead to immune cascades that re-ignite inflammation.

We found that during DSS colitis, both conventional T cells and Tregs were generated against oral antigens, while healthy mice only developed OVA-reactive Tregs. Classically, exposure to oral antigens leads to Foxp3+ Treg responses that control untoward responses to microbiota and food antigens that are induced via CD103+ DCs producing TGFβ, retinoic acid and prostaglandins [23]. However, in the DSS model of colitis, the weakening of the mucus barrier allows the penetration of bacteria to the underlying immune cells [17]. This likely leads to the release of an abundance of pro-inflammatory cytokines [33], and this would allow the generation of other non-regulatory CD4+ T cell effector subsets. This concept was supported by our ability to only detect OVA-directed conventional T cells in DSS-treated mice.

We were only able to find oral antigen reactive T cells and cytokine-producing effector T cells within the spleen and not the mLN. Literature supports this observation as T cells are known to travel to the spleen after the resolution of acute inflammation [30]. Moreover, Hall et al. demonstrated that after resolution of acute DSS colitis (day 25 after the start of DSS), there is a striking increase of activated CD4^+^ T cells in the spleen, while the percentage of activated T cells within the mLN normalizes [34]. We cannot eliminate the possibility that examination of mLNs using a more refined technique, such as tetramer staining, may reveal the presence of OVA-reactive T cells. However, despite lack of sensitivity, it is clear that OVA-directed responses are more pronounced in the spleen at the time point that we tested.

To our knowledge, we have demonstrated for the first time that oral antigen-specific T cells form during DSS colitis and that they can be found systemically after the resolution of colitis. This gives added depth and usefulness to the DSS colitis model, specifically as oral antigens can now be considered as a strategy to track T cells primed by gut luminal antigens. This new perspective on the DSS-induced colitis model will allow insight into the inflammatory processes needed to initiate the development of antigen-specific T cells during acute gastrointestinal inflammation and their role in disease chronicity.
